# Comparative evaluation of intravenous versus intranasal dexmedetomidine on emergence delirium and hemodynamics in pediatric patients undergoing adenotonsillectomy: a randomized controlled trial

**DOI:** 10.3389/fphar.2025.1543344

**Published:** 2025-01-30

**Authors:** Chenxu Dai, Xuemei Zhao, Aoxue Li, Xuedong Zhang, Penglei Wang, Ye Zhang, Yun Wu

**Affiliations:** ^1^ Department of Anesthesiology and Perioperative Medicine, The Second Affiliated Hospital of Anhui Medical University, Hefei, China; ^2^ Department of Anesthesiology, Fuyang People’s Hospital, Fuyang, China; ^3^ Department of Anesthesiology, Children’s Hospital of Anhui Province, Hefei, China

**Keywords:** dexmedetomidine, emergence delirium, intravenous, intranasal, hemodynamics, recovery

## Abstract

**Background:**

Dexmedetomidine effectively prevents emergence delirium in children. However, intravenous dexmedetomidine is frequently associated with hemodynamic instability and delayed recovery. Intranasal dexmedetomidine has been proposed as a method of reducing these side effects. This study aimed to evaluate the effects of intranasal versus intravenous dexmedetomidine on emergence recovery and hemodynamics in children undergoing adenotonsillectomy.

**Methods:**

A total of 139 children, aged 3–10 years, who were scheduled for elective adenotonsillectomy were randomly assigned to receive intravenous dexmedetomidine (IV DEX group) or intranasal dexmedetomidine (IN DEX group), or saline (control group) after anesthesia induction. The primary outcome was the highest score on the pediatric anesthesia emergence delirium (PAED) score during the first 30 min after awakening. Secondary outcomes included the perioperative blood pressure and heart rate, time to awakening, postoperative pain score, and length of post-anesthesia care unit (PACU) stay.

**Results:**

The highest PAED and pain scores were significantly lower in the IV and IN DEX groups than those in the control group during the first 30 min after awakening. However, no significant differences were observed between the IV and IN DEX groups. Notably, patients in the IN DEX group exhibited a significantly lower PAED score at 2 h and lower pain scores at 2, 4, and 6 h postoperatively than those in the IV DEX group. Patients in the IV DEX group exhibited a significantly longer awakening time and length of PACU stay than those in the IN DEX and control groups. In the IV DEX group, the heart rate was significantly lower perioperatively than at baseline, while this effect was not observed in the IN DEX group.

**Conclusion:**

Both intravenous and intranasal administration of dexmedetomidine after induction of anesthesia effectively improved emergence delirium and pain intensity in children undergoing adenotonsillectomy. Intranasal administration of dexmedetomidine provided more stable hemodynamics and more prolonged analgesia and sedation than intravenous infusion of dexmedetomidine.

**Clinical Trial Registration:**

https://www.chictr.org.cn/showproj.html?proj=180658.

## Introduction

Emergence delirium (ED) is a common complication that occurs frequently in children, with a reported incidence of 10%–80% (Moore and Anghelescu, 2017; Keaney et al., 2004). ED has been described as a mental disturbance, dissociated state of consciousness, or state of confusion without recognition of the surrounding environment. It is accompanied by agitation behaviors such as kicking, screaming, thrashing, or involuntary physical activity, which not only affect the quality of recovery but increase the risk of self-injury, surgical dehiscence, sleeping disorders, enuresis, and even persistent changes in emotional and cognitive function (Manning et al., 2020; Hauber et al., 2015). Adenotonsillectomy is a risk factor for postoperative ED in pediatric surgery (He et al., 2023). Various sedative and analgesic agents administrated systemically are efficient in preventing ED after adenotonsillectomy.

Dexmedetomidine, a highly selective short-acting, alpha2-adrenoreceptor agonist, has sedative, analgesic, and anxiolytic properties without respiratory depression. The intravenous (IV) infusion of dexmedetomidine of varying dosages and time courses, has been shown to be effective in reducing the incidence and severity of ED in children (Shi et al., 2019; Guler et al., 2005; Oğurlu et al., 2010). However, adverse hemodynamic complications such as hypotension and bradycardia might hinder its widespread use in these patients. IV dexmedetomidine is also associated with delayed recovery and a prolonged post-anesthesia care unit (PACU) stay, owing to its sedative effects (Manning et al., 2020; Zhu et al., 2015). In minor procedures of brief duration such as adenotonsillectomy, it is important to explore strategies that can prevent postoperative ED and improve the quality of children’s postoperative condition, without increasing anesthesia-related complications and prolonged PACU stay.

Alternatives to rapid IV delivery have been proposed as methods of reducing the side effects of dexmedetomidine (Niyogi et al., 2019). Dexmedetomidine is also effective when administered orally, intramuscularly, and intranasally. Intranasal (IN) dexmedetomidine, a more practical and efficient delivery method, has recently been shown to have positive perioperative outcomes in multiple studies of pediatric patients (Niyogi et al., 2019; M and Nelamangala, 2023). However, to our knowledge, few studies have compared the efficacy of perioperative IV dexmedetomidine with IN dexmedetomidine in preventing ED in pediatric patients. This study was designed to evaluate the sedative and hemodynamic effects and recovery after IV and IN administration of dexmedetomidine to prevent ED in children undergoing adenotonsillectomy. The primary outcome was the highest ED score evaluated using the Pediatric Anesthesia Emergence Delirium (PAED) score (Wang et al., 2021) during the first 30 min after awakening. We hypothesized that IN dexmedetomidine would improve the ED with lower anesthetic-related adverse events. In addition, the incidence of ED, pain score, incidence of rescue analgesic use, hemodynamic parameters, adverse events, awakening time, length of PACU stay, and postoperative sedation were also assessed.

## Material and methods

### Study design and randomization

This trial was performed at the Children’s Hospital of Anhui Province, Hefei, China, between 12 November 2022, and 10 September 2023. The study protocol was approved by the Medical Ethics Committee of the Children’s Hospital of Anhui Province (approval no: EYLL-2022-023) and registered at the Chinese Clinical Trial Registration Center (http://www.chictr.org.cn; ChiCTR2200065404) on 03 November 2022. The study was performed in accordance with the Consolidated Standards of Reporting Trials (CONSORT) criteria (Schulz et al., 2010) and complied with the Declaration of Helsinki. After taking assent from the enrolled children and the guardians, written informed consent was obtained from the guardians of the participants.

Pediatric patients aged 3–10 years with an American Society of Anesthesiologists physical status of I or II who were scheduled for adenotonsillectomy requiring general anesthesia were enrolled. Children with a body mass index (BMI) > 25 kg/m^2^; known cardiopulmonary, liver, or kidney disease; developmental delays; psychological or neurological disorders; abnormal airways; reactive airway disease; history of general anesthesia; history of previous allergies or known allergies to the current study’s drugs; history of chronic pain or recent administration of sedative and analgesic drugs; and parents or guardians who refused to allow their children to participate, were excluded from this study.

The enrolled patients were randomly assigned to the control group, IV dexmedetomidine group (IV DEX group), or IN dexmedetomidine group (IN DEX group) using SPSS (version 26.0; IBM, Armonk, NY) (Li et al., 2020) in a 1:1:1 ratio. An assistant who was not involved in the children’s clinical management or data collection performed the blinded random allocation by preparing coded and sealed opaque envelopes. A nurse unaffiliated to patient care opened the envelopes shortly before induction and prepared the study medications outside the operating room. The agent used for this study was diluted with NaCl (0.9%) to yield two study drug syringes: a 1-mL syringe containing either 100 μg mL^-1^ dexmedetomidine hydrochloride (Hengrui Pharmaceutical Co., LTD., China) or NaCl (0.9%) and a 50-mL syringe containing either 4 μg mL^-1^ dexmedetomidine hydrochloride or NaCl (0.9%), which were identical in appearance and were labeled as “study medication” with the patient number. Thereafter, the patients, anesthesiologists, nurses providing postoperative care, surgeons, investigators, and outcome assessors were blinded to the patients’ group allocations and did not have access to randomization until the data analysis was complete.

### Study procedures and interventions

All patients fasted for 8 h with the opportunity to drink clear fluids up to 3 h before surgery. The participants stayed with a caregiver in the pre-anesthesia room without premedication. Preoperative anxiety was assessed using the Parental Separation Anxiety Scale (PSAS), a four-point behavior score: 1 = calm and cooperative, 2 = anxious but reassurable, 3 = anxious and not reassurable, and 4 = crying or resisting (Chen et al., 2023; Cho et al., 2020). After pulse oximetry was monitored, all children received anesthesia induction in the presence of their caregivers. Anesthesia was induced by inhalation of 8% sevoflurane with an oxygen inflow of 8 L/min using a face mask. Once consciousness was lost, sevoflurane was adjusted to 3%–4% with an oxygen inflow of 2 L/min, and IV access was established. The children were separated from their caregivers, transferred to the operating room, and monitored using noninvasive arterial pressure, pulse oximetry, capnography, and electrocardiography throughout the surgery. All patients received antiemetics with dexamethasone sodium phosphate (Hainan Best Pharmaceutical Co., LTD., China) 0.15 mg/kg intravenously to prevent postoperative nausea and vomiting, and penehyclidine hydrochloride (Nhua Pharmaceutical Co., LTD., China) 0.01 mg/kg to prevent glandular secretion. Endotracheal intubation was then facilitated using IV sufentanil 0.2 μg/kg and cisatracurium 0.1 mg/kg–0.2 mg/kg. Children were mechanically ventilated using volume-controlled ventilation. The tidal volume was set to 6–8 mL/kg, while the respiratory rate was set to 16 beats/min and further adjusted to maintain the end-tidal carbon dioxide pressure between 35 and 45 mmHg.

After intubation, patients received interventions according to their allocation. Patients in the IV DEX group received IN NaCl (0.9%) and IV dexmedetomidine at 1 μg/kg for 10 min; patients in the IN DEX group received IN dexmedetomidine at 2 μg/kg and IV NaCl (0.9%) for 10 min; patients in the control group received both IN and IV NaCl (0.9%). For the administration of IN dexmedetomidine, the prepared drug solution was administered dropwise in both nostrils using a needleless 1-mL syringe, with the head turned to the side, allowing the drug to stay in contact with the lateral nasal mucosa. If nasal secretions were visible, the nostrils were suctioned before drug administration.

All patients underwent the Coblation adenotonsillectomy. After placing a McIvor mouth gag in the oral cavity, a rubber catheter was inserted through the nasal cavity and out the oropharynx to retract the soft palate. A dental mirror then was used for direct visualization of the surgical site. The coblation procedure was performed using an Evac 70 Arthro Wand (Arthro Care Corp. Sunnyvale, CA) and bleeding secured with coblation (Mularczyk et al., 2018; Paramasivan et al., 2012). Anesthesia was maintained by inhalation of sevoflurane 2%–3%, which was discontinued approximately 5 min before the completion of surgery. Additionally, IV propofol (2–4 mg/kg/h) and remifentanil (0.1–0.2 μg/kg/min) were infused continuously until surgery was complete. After surgery, children with a tracheal tube were transferred to the PACU. After confirming regular breathing with sufficient tidal volume (>5 mL/kg) and SpO_2_ > 95%, without the need for oxygen supplementation, tracheal extubation was performed under a deep level of sedation. After tracheal extubation, oxygen was provided with an inflow of 2 L/min by using a nasal cannula.

Immediately after tracheal extubation, an investigator who was blinded to the group allocation evaluated and recorded the degree of delirium, postoperative pain intensity, and level of sedation every 10 min during the first 30 min of the emergence period. Delirium was evaluated using the PAED score, which consists of five psychometric items describing emergence behavior, with scores ranging from 0 to 20. The final PAED score was derived by adding the scores for each item, with higher scores indicating a higher degree of delirium (Supplementary Table S1). ED was defined as a PAED score ≥12 (Shi et al., 2019); participants with scores ≥12 for >5 min were treated with IV propofol 1 mg/kg as a rescue medication. Postoperative pain intensity was assessed every 10 min using the modified Children’s Hospital of Eastern Ontario Pain Scale (mCHEOPS). The mCHEOPS score is based on five items, including crying, facial expression, verbal responses, torso movements, and leg position, and ranges from 0 to 10 (Hong et al., 2017) (Supplementary Table S2). Participants with mCHEOPS scores of ≥5 were administered IV sufentanil 2 μg. The sedation level was assessed using the Ramsay sedation scale (Heavner et al., 2022) (Supplementary Table S3).

All children were transferred to the ward when they became calm and met the modified Aldrete score of ≥9 (Aldrete, 2007) (Supplementary Table S4). If the patients required additional pain relief, two puffs of lidocaine spray (about 30 mg, Xiangxue Pharmaceutical Co. LTD., China) was sprayed to the pharynx of patients, while no intravenous analgesic was administered. Patients were monitored overnight, and discharged with stable vital signs and with no evidence of complications in the morning of the first postoperative day. Patients were followed-up during the first 24 h post-surgery. The degree of delirium, postoperative pain intensity, and sedation level were also recorded at 2, 4, 6, and 8 h after surgery, as were cardiovascular and respiratory adverse events such as airway complications, oxygen desaturation (defined as SpO_2_ <90%), or bleeding that occurred postoperatively.

### Outcome measures

Baseline data included patient characteristics and preoperative PSAS score. Intraoperative data included duration of surgery and anesthesia (defined as the interval between the beginning of anesthesia induction and discontinuation of the anesthetics). The primary outcome was the highest PAED score recorded during the first 30 min after awakening in the PACU. The secondary outcomes included the perioperative mean arterial pressure (MAP) and heart rate (HR), time to extubation (defined as the interval between discontinuation of the anesthetics and extubation), time to awakening (defined as the interval between discontinuation of the anesthetics and eyes opening by request), incidence of PAED score ≥12, mCHEOPS score, length of PACU stay, and incidences of adverse events.

### Sample size and statistical analysis

The sample size was calculated based on the primary outcome (highest PAED score during the first 30 min of emergence) using PASS software (version 15.0; NCSS Statistical Software, LLC, Kaysville, Utah, United States). Based on the results of our pilot study with six patients in each group, wherein the primary outcome scores (means ± standard deviations [SD]) were 7.2 ± 1.3 for the IV DEX group, 9.7 ± 3.0 for the IN DEX group, and 12.2 ± 2.9 for the control group, we set the minimum detectable difference between groups at 2.5, using a pooled SD of 2.3. One-way analysis of variance (ANOVA) with multiple comparisons was selected and grouped into three groups; the group allocation ratios were equal. At a power of 0.80 and an alpha error of 0.05, to account for 10% loss to follow-up, the required sample size for each group was calculated as 47. Thus, 141 participants were included in this study.

All statistical analyses were performed using SPSS (version 26.0; IBM Corp., Armonk, NY, United States). The Kolmogorov–Smirnov test and visual inspection of the histograms were performed to assess data normality. Continuous variables were expressed as mean (SD) or median (interquartile range, IQR), and inter-group differences were assessed for significance using ANOVA for normally distributed data or the Kruskal–Wallis test for nonparametric data followed by Bonferroni correction. Categorical variables were expressed as numbers (percentages), and inter-group differences were assessed using chi-squared or Fisher’s exact tests in cases of expected frequency <5. Repeated measurements of intraoperative hemodynamic parameters and postoperative PAED, pain, and sedation scores were analyzed using a linear mixed model to evaluate the association between the dependent variables over time and dexmedetomidine administration. *P*-values were corrected using Bonferroni correction (adjusted by multiplying by the number of tests).

## Results

**FIGURE 1:**
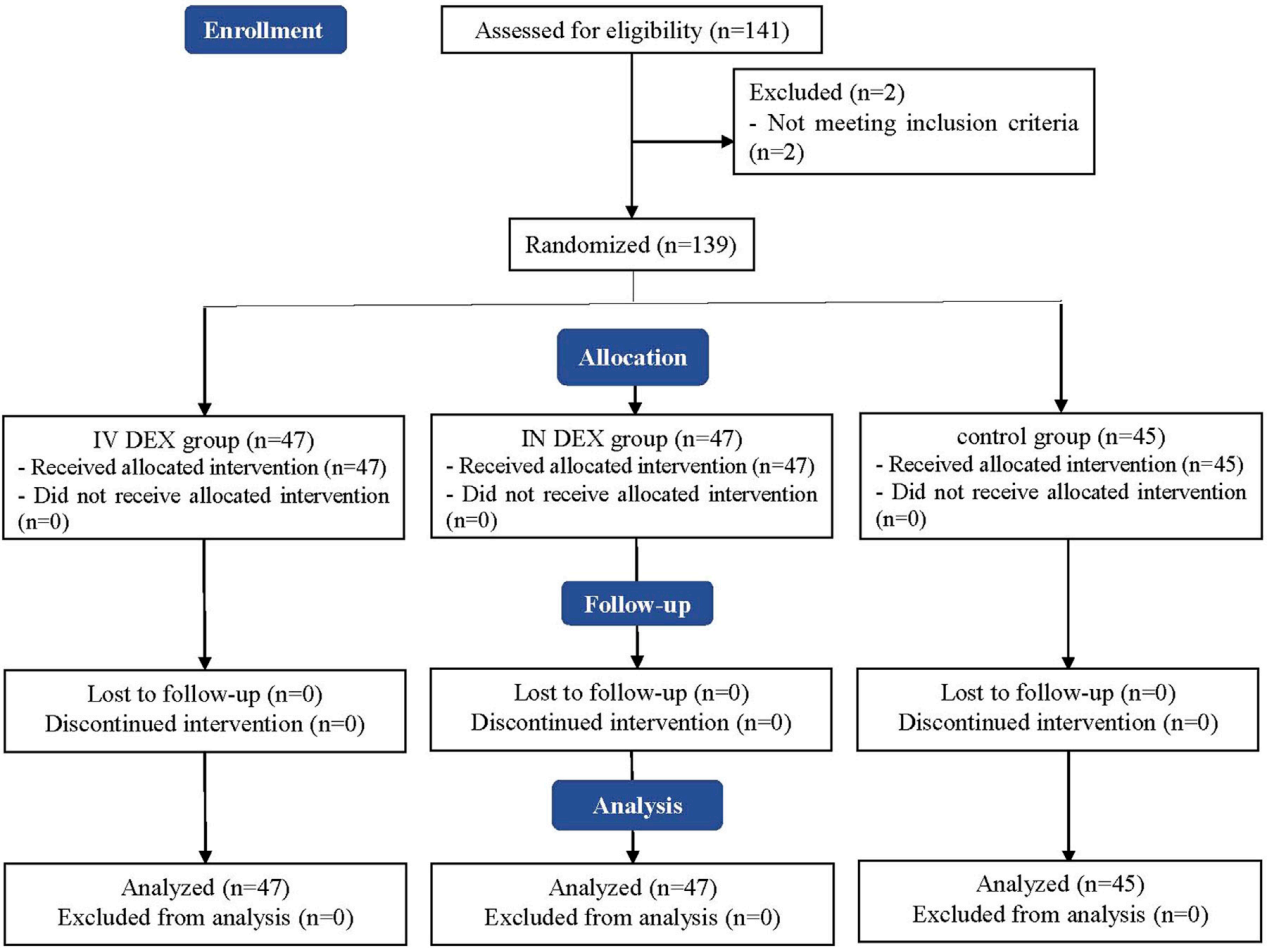
Consolidated Standards of Reporting Trials (CONSORT) flowchart of patient enrollment, allocation, follow-up, and analysis. IV DEX, intravenous dexmedetomidine group; IN DEX, intranasal dexmedetomidine group.

A CONSORT flow diagram of this trial is shown in Figure 1. A total of 141 children were initially screened for suitability, and two children did not meet our inclusion criteria because of cardiac disease. Ultimately, 139 children were enrolled and randomized, all of whom were followed-up until the end of the study.

Patient characteristics and perioperative parameters are shown in Table 1. No significant differences were observed in the overall characteristics, preoperative PSAS scores, duration of anesthesia, or surgery among the three groups.

**TABLE 1:** Patients’ characteristics and perioperative parameters.

Parameters	IV DEX (n = 47)	IN DEX (n = 47)	Control (n = 45)	*P* value
Age, y	5.9 (1.5)	6.0 (1.5)	5.9 (1.5)	0.970
Sex, male	27 (57.4)	32 (68.1)	28 (62.2)	0.566
Body mass index, kg/m^2^	16.2 (2.1)	16.5 (2.1)	17.0 (1.8)	0.169
PSAS score	1.0 (1.0, 2.0)	1.0 (1.0, 2.0)	1.0 (1.0, 2.0)	0.868
Duration of surgery, min	30.9 (5.6)	30.9 (6.9)	31.0 (4.7)	0.987
Duration of anesthesia,^a^ min	39.8 (6.0)	38.1 (6.6)	39.4 (4.8)	0.367

Data are presented as mean (standard deviation), median (interquartile range) or n (%). Intergroup differences were assessed for significance using ANOVA or chi-squared tests, and pairwise comparisons were analyzed using Bonferroni correction.

^a^ Duration of anesthesia is defined as the interval between the beginning of anesthesia induction and discontinuation of the anesthetics. IV DEX, intravenous dexmedetomidine group; IN DEX, intranasal dexmedetomidine group; PSAS, parental separation anxiety scale.

**FIGURE 2:**
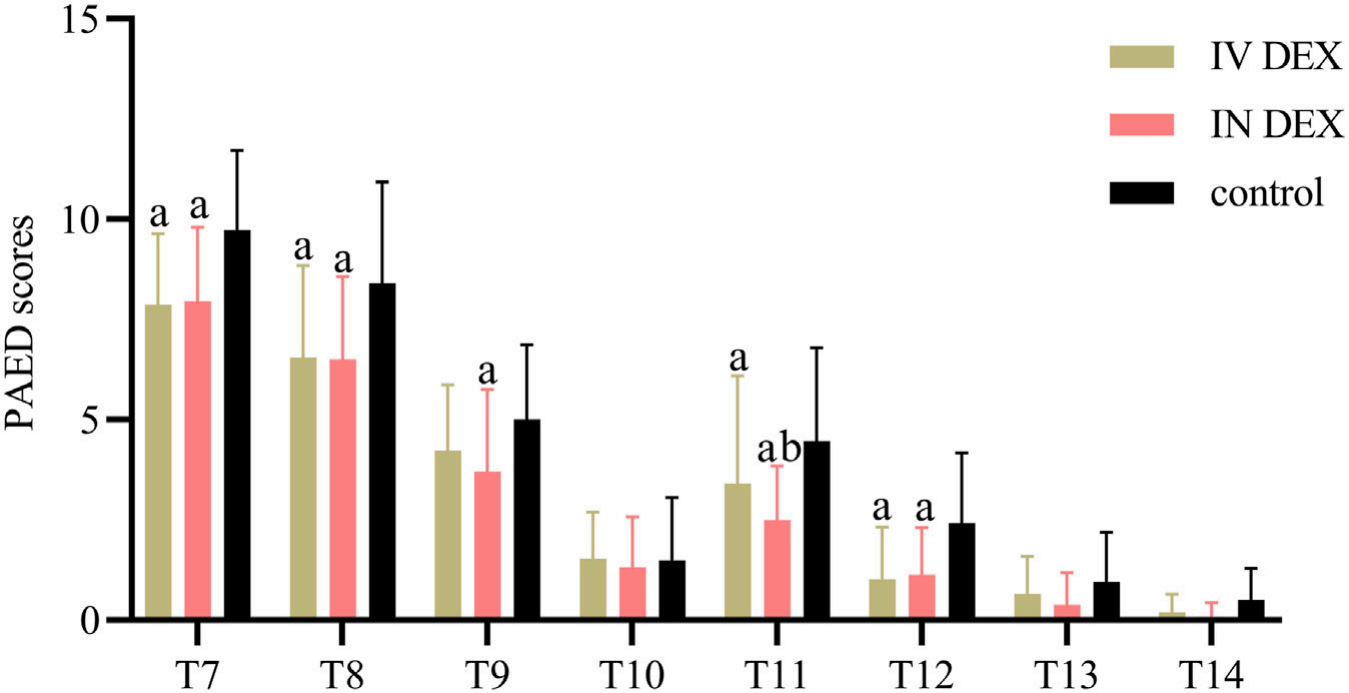
PAED sores after surgery. Data are expressed as mean (standard deviation). Comparisons between groups were made using linear mixed-model analyses. ^a^ *P* < 0.05 indicates statistically significant differences compared with the control group; ^b^ *P* < 0.05 indicates statistically significant differences compared with the IV DEX group. *P* values are corrected using Bonferroni correction. T7, At wakening; T8, 10 min after awakening; T9, 20 min after awakening; T10, 30 min after awakening; T11, 2 h after surgery; T12, 4 h after surgery; T13, 6 h after surgery; T14, 8 h after surgery; IV DEX, intravenous dexmedetomidine group; IN DEX, intranasal dexmedetomidine group; PAED pediatric anesthesia emergence delirium.

**FIGURE 3:**
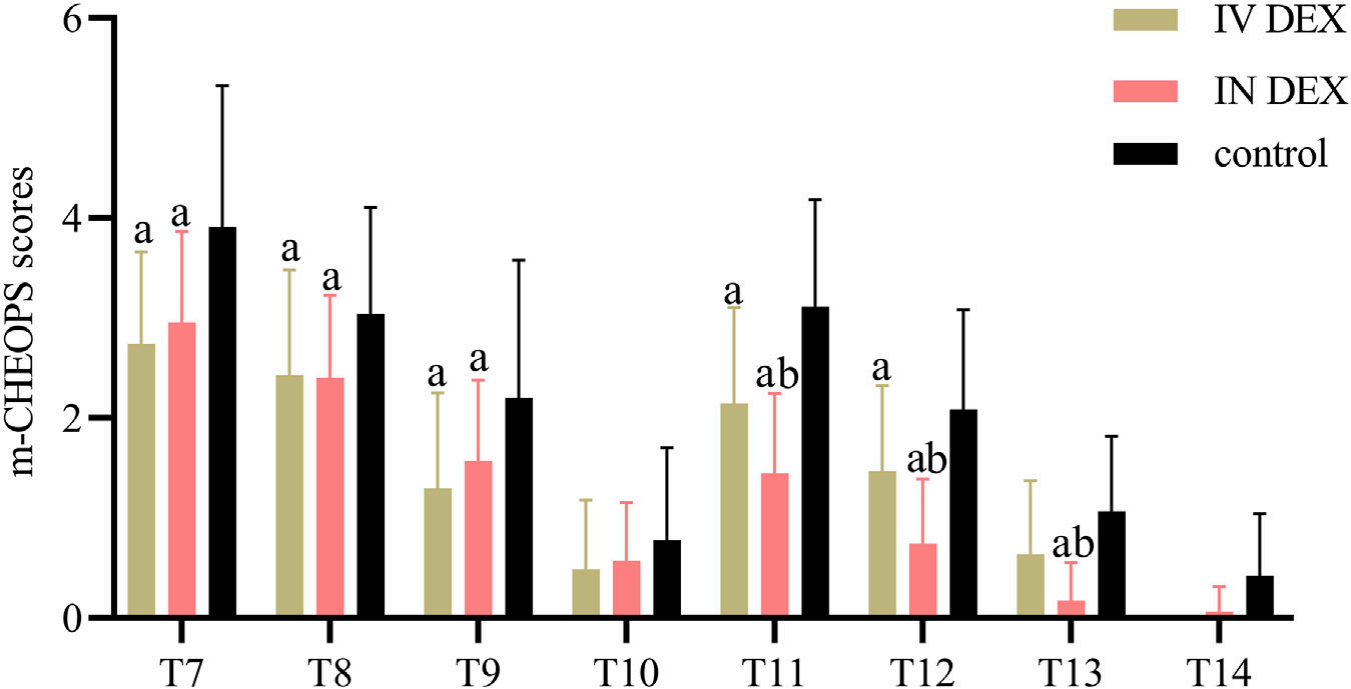
m-CHEOPS scores after surgery. Data are expressed as mean (standard deviation). Comparisons between groups were made using linear mixed-model analyses. ^a^ *P* < 0.05 indicates statistically significant differences compared with the control group; ^b^ *P* < 0.05 indicates statistically significant differences compared with the IV DEX group. *P* values are corrected using Bonferroni correction. T7, At wakening; T8, 10 min after awakening; T9, 20 min after awakening; T10, 30 min after awakening; T11, 2 h after surgery; T12, 4 h after surgery; T13, 6 h after surgery; T14, 8 h after surgery; IV DEX, intravenous dexmedetomidine group; IN DEX, intranasal dexmedetomidine group; m-CHEOPS, modified children’s hospital of eastern ontario pain scale.

**FIGURE 4:**
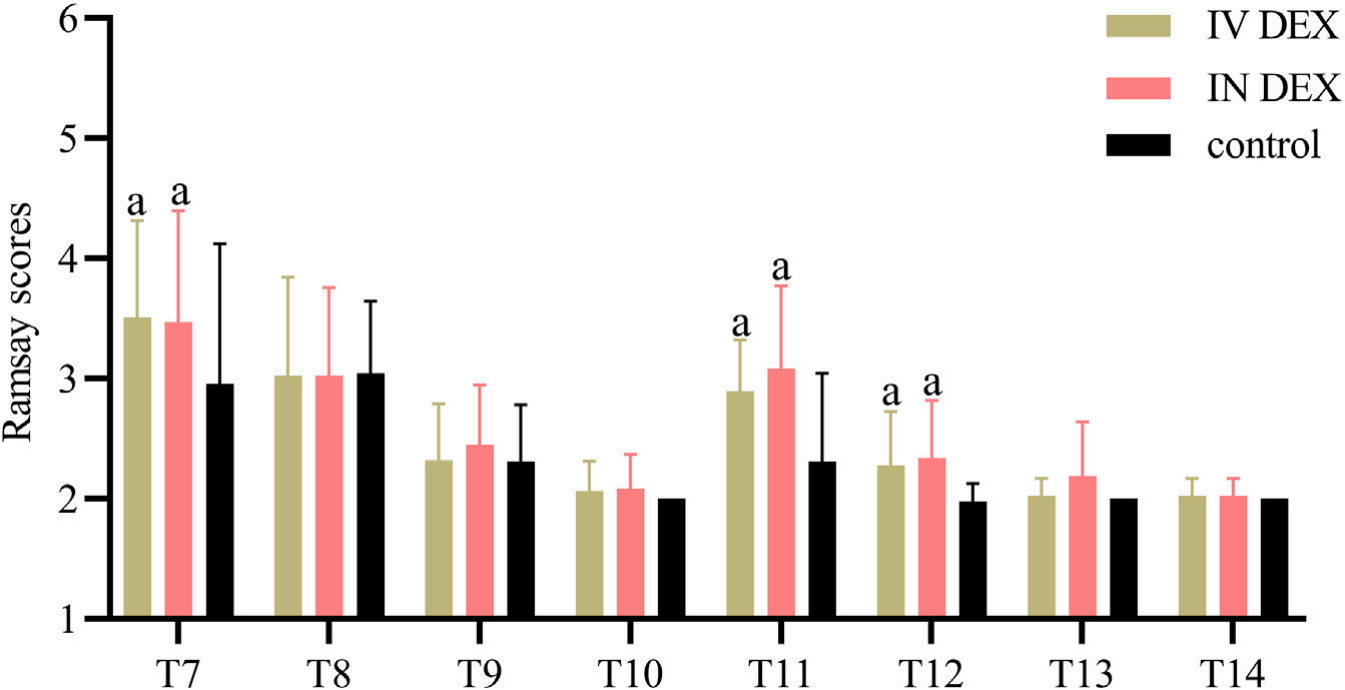
Ramsay scores after surgery. Data are expressed as mean (standard deviation). Comparisons between groups were made using linear mixed-model analyses. ^a^ *P* < 0.05 indicates statistically significant differences compared with the control group. *P* values are corrected using Bonferroni correction. T7, At wakening; T8, 10 min after awakening; T9, 20 min after awakening; T10, 30 min after awakening; T11, 2 h after surgery; T12, 4 h after surgery; T13, 6 h after surgery; T14, 8 h after surgery; IV DEX, intravenous dexmedetomidine group; IN DEX, intranasal dexmedetomidine group.

The mean (SD) of the highest PAED scores after awakening differed significantly among the three groups (IV DEX group, 8.3 [2.2]; IN DEX group, 8.3 [2.1]; control group, 10.3 [2.2]; *P* < 0.001). The differences were statistically significant for the IV DEX vs control group (mean difference = −2.0, 95% CI = −3.1 to −0.9, *P* < 0.001) and IN DEX vs control group (mean difference = −2.0, 95% CI = −3.1 to −0.9, *P* < 0.001) but not for the IV vs IN DEX group (mean difference = −0.0, 95% CI = −1.1 to 1.1, *P* > 0.999) (Table 2). In addition to the highest scores, patients in the IV and IN DEX groups exhibited significantly lower PAED scores than those in the control group during the first 20 min after awakening. No significant differences were observed between the IV and IN DEX groups. In the ward, the PAED scores were significantly lower in the two dexmedetomidine groups at 2 and 4 h after surgery than in the control group. When compared between the two dexmedetomidine groups, the PAED score was significantly lower in the IN DEX group than in the IV DEX group at 2 h after surgery (Figure 2). Similarly, the highest mCHEOPS scores were significantly lower in the IV and IN DEX groups than in the control group (IV DEX vs control group, mean difference = −1.3, 95% CI = −1.9 to −0.6, *P* < 0.001; IN DEX vs control group, mean difference = −1.1, 95% CI = −1.7 to −0.5, *P* < 0.001). The highest mCHEOPS scores were comparable between the two dexmedetomidine groups (mean difference = −0.1, 95% CI = −0.8 to 0.5, *P* > 0.999) (Table 2). Moreover, patients in the IV and IN groups exhibited significantly lower mCHEOPS scores than those in the control group during the first 20 min after awakening and until 6 h after surgery in the ward. Notably, the mCHEOPS scores were significantly lower in the IN DEX group than in the IV DEX group at 2, 4, and 6 h after surgery (Figure 3). Patients receiving IV and IN dexmedetomidine exhibited significantly higher sedation scores at the time of awakening and at 2 and 4 h after surgery than those in the control group. However, none of the patients developed airway complications or oxygen desaturation (Figure 4).

**TABLE 2:** The highest value of PAED and m-CHEOPS scores within 30 min after awakening.

	IV DEX (n = 47)	IN DEX (n = 47)	Control (n = 45)	Mean difference (95% CI) of pairwise comparisons *P* value		
				IV DEX vs. IN DEX	IV DEX vs. control	IN DEX vs. control
PAED scores	8.3 (2.2)	8.3 (2.1)	10.3 (2.2)	−0.0 (−1.1 to 1.1)	−2.0 (−3.1 to −0.9)	−2.0 (−3.1 to −0.9)
				>0.999	<0.001	<0.001
m-CHEOPS scores	2.9 (1.1)	3.0 (1.0)	4.1 (1.6)	−0.1 (−0.8 to 0.5)	−1.3 (−1.9 to −0.6)	−1.1 (−1.7 to −0.5)
				>0.999	<0.001	<0.001

Data are presented as mean (standard deviation), intergroup differences were assessed for significance using ANOVA, and pairwise comparisons were analyzed using Bonferroni correction. IV DEX, intravenous dexmedetomidine group; IN DEX, intranasal dexmedetomidine group; CI, confidence interval; PAED, pediatric anesthesia emergence delirium; m-CHEOPS, modified children’s hospital of eastern ontario pain scale.

No significant differences were observed in the time to extubation or incidence of rescue analgesia among the three groups. The IN DEX groups had a significantly lower incidence of rescue sedation than the control group. No significant differences were observed between the IV and IN DEX groups, or IV DEX and control groups. Patients in the IV DEX group exhibited a significantly longer awakening time and length of PACU stay than those in the IN DEX and control groups. The time to awakening and PACU stay were similar between the IN DEX and control groups (Table 3).

**TABLE 3:** Postoperative conditions in three groups.

Parameters	IV DEX (n = 47)	In DEX (n = 47)	Control (n = 45)	*P* value
Time to awakening,^c^ min	39.0 (4.3)^a^	23.8 (4.8)^b^	22.0 (7.0)	<0.001
Time to extubation,^d^ min	12.4 (2.1)	12.5 (2.5)	12.8 (1.7)	0.595
Length of PACU stay, min	53.0 (3.8)^a^	39.3 (3.2)^b^	37.6 (4.6)	<0.001
Incidence of rescue analgesia	1 (2.1)	2 (4.3)	7 (15.6)	0.052
Incidence of rescue sedation	6 (12.8)	5 (10.6)^a^	14 (31.1)	0.020

Data are presented as mean (standard deviation) or n (%). Intergroup differences were assessed for significance using ANOVA or chi-squared tests were analyzed using Bonferroni correction.

^a^ *P* < 0.05, indicates statistically significant differences compared with the control group; ^b^ *P* < 0.05 indicates statistically significant differences compared with the IV DEX group. IV DEX, intravenous dexmedetomidine group; IN DEX, intranasal dexmedetomidine group; PACU, post-anesthesia care unit.

^c^ Time to awakening is defined as the interval between discontinuation of the anesthetics and eyes opening by request.

^d^ Time to extubation is defined as the interval between discontinuation of the anesthetics and extubation.

**FIGURE 5:**
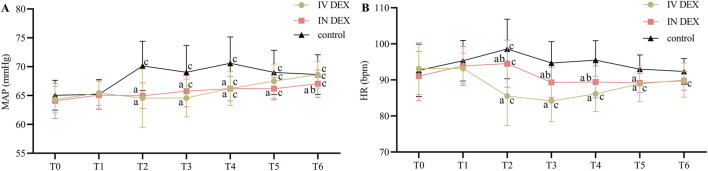
Perioperative MAP **(A)** and HR **(B)** values. Data are expressed as mean (standard deviation). Comparisons between groups were made using linear mixed-model analyses. ^a^ *P* < 0.05 indicates statistically significant differences compared with the control group; ^b^ *P* < 0.05 indicates statistically significant differences compared with the IV DEX group; ^c^ *P* < 0.05 indicates statistically significant differences compared with the baseline value. T0, Before anesthesia (baseline); T1, Before intervention; T2, 10 min after intervention; T3, Tracheal extubation; T4, 10 min after tracheal extubation; T5, 20 min after tracheal extubation; T6, 30 min after tracheal extubation. IV DEX, intravenous dexmedetomidine group; IN DEX, intranasal dexmedetomidine group.

The MAP and HR were comparable among the three groups before anesthesia and intervention. However, they were significantly lower in the IV and IN DEX groups than in the control group after drug administration, at the time of tracheal extubation, and during the PACU stay. In the IV DEX group, the HR was significantly lower after the administration of dexmedetomidine, at tracheal extubation and in the PACU than at baseline, while this effect was not observed in the IN DEX group (Figures 5A, B).

## Discussion

Our data revealed that compared with saline infusion, both IV and IN administration of dexmedetomidine after anesthesia induction were effective in reducing the severity and incidence of ED and pain intensity in children undergoing adenotonsillectomy. However, IV infusion of dexmedetomidine with 1 μg/kg was associated with a lower HR and delayed recovery. IN dexmedetomidine with 2 μg/kg provided more stable hemodynamics during anesthesia, and more prolonged analgesia and sedation in the ward than IV dexmedetomidine.

Adenotonsillectomy is a risk factor for pediatric ED because of the postoperative pain, feeling of suffocation, and bleeding. In addition to providing sedation and anxiolysis, dexmedetomidine has analgesic properties and reduces stress responses during surgical procedures. It efficiently prevents ED and pain intensity in both adults and children (Shi et al., 2019; Shin et al., 2023). In previous studies using dexmedetomidine for the prevention of ED in children, the loading dosage of IV infusion varied from 0.3 to 2.5 μg/kg (Jain et al., 2018; Rao et al., 2020). Consistently, we demonstrated that 1 μg/kg of dexmedetomidine administered intravenously after induction resulted in significantly lower PAED and pain scores after adenotonsillectomy than those achieved using saline. However, the potential hemodynamic effects of dexmedetomidine sedation are a key concern. Bradycardia and hypotension are the most frequently reported adverse hemodynamic effects of dexmedetomidine, owing to its peripheral sympatholytic properties (Weerink et al., 2017). The incidence of bradycardia in pediatric patients has been as high as 16% at the loading dose of 2–3 μg/kg (Mason et al., 2008). Because the dexmedetomidine-induced reduction in HR was shown to exhibit a dose-response relationship (Ebert et al., 2000), we selected a relatively lower dose of dexmedetomidine at 1 μg/kg to avoid the incidence of hemodynamic instability. Although no bradycardia events occurred in our study, the children still exhibited a significantly reduced HR after receiving IV dexmedetomidine, which might have increased the risk of bradycardia and hypotension.

IN administration is a convenient, simple, non-invasive approach of administration that reduces first-pass effects and has been successfully applied to dexmedetomidine. IN administration of dexmedetomidine has recently been shown to have positive perioperative outcomes in multiple studies in children and the dose could be tolerated safely in high doses up to 4 μg/kg (El-Hamid and Yassin, 2017; Yuen et al., 2008; Tug et al., 2015; Yuen et al., 2012). Iirola et al. found that the absolute bioavailability of IN dexmedetomidine was 65% (35%–93%) (Iirola et al., 2011). Because younger children may be frightened or excessively anxious when roused and awoken in a strange environment, especially children in whom communication is difficult, a relatively deep level of sedation should be chosen to facilitate a smooth recovery. Therefore, patients in the IN DEX group received a dosage of 2 μg/kg of dexmedetomidine after induction. As in the IV DEX group, the administration of IN dexmedetomidine also resulted in better ED and pain intensity after adenotonsillectomy than those after saline administration. Moreover, IN administration prevented acute perioperative hemodynamic changes, as patients receiving IN dexmedetomidine exhibited more stable hemodynamics than those in the IV DEX group. A recently published meta-analysis documented that the incidence of bradycardia associated with the IV, intramuscular, and IN routes was approximately 5%, 2%, and 0.002%, respectively (Gong et al., 2017). We speculated that the minor effect on the HR might be due to the absorption phase of IN dexmedetomidine. IN dexmedetomidine is associated with a slower and more gradual onset than IV dexmedetomidine. On average, peak plasma concentrations of dexmedetomidine were obtained within 30 min of IN administration, although the time to peak concentration varied widely (Yoo et al., 2015). In a study comparing IV 1 μg/kg dexmedetomidine with IN 1 μg/kg dexmedetomidine, the onset times were 15–20 and 30–45 min, respectively (Zhang et al., 2013). A more gradual onset may actually be desirable to avoid the high peak plasma levels and the cardiovascular inhibition effects, including bradycardia, observed with rapid IV administration (Niyogi et al., 2019). Thus, hemodynamic alterations were less severe in the IN DEX group than in the IV DEX group, with lower plasma concentrations.

Delayed recovery with IV dexmedetomidine has also been documented owing to its sedative effects (Chen et al., 2014). Alternative routes other than rapid IV delivery may help to minimize the adverse effects of dexmedetomidine (Iirola et al., 2011). In the current study, patients were extubated under deep sedation. Owing to the lack of respiratory depressive action, dexmedetomidine administration did not delay the extubation times. However, patients receiving IV dexmedetomidine exhibited a longer awakening time. Meanwhile, the decrease of HR during emergence recovery led to prolonged monitoring of the patients, which made their length of PACU stay longer than those in the control group. Notably, no significant differences were observed between the IN DEX and control groups. We also found that patients receiving IN dexmedetomidine exhibited lower PAED and pain scores than those receiving IV dexmedetomidine until 6 h after surgery. This prolonged efficiency might attribute to the delayed absorption of IN route. Yuen et al. demonstrated that IN dexmedetomidine at 1–1.5 μg/kg produced sedation in 45–60 min and peaks in 90–105 min (Yuen et al., 2007). From this point of view, IN dexmedetomidine with 2 μg/kg might provide extended duration of action for prevention of ED in the ward.

Although we observed the positive effects of IN dexmedetomidine in children, our study has several limitations. First, the plasm concentration, pharmacodynamics and pharmacokinetics of dexmedetomidine were not tested, we were not certain about the exact dosage patients finally received; Second, the dosage of dexmedetomidine originated from previous literature and our clinical practice; we acknowledge that it was not optimal. Stable hemodynamics might be achieved with a lower loading dosage of dexmedetomidine. Thus, a dose-comparison study is warranted in the future. Third, since the concentration of the original dexmedetomidine was 100 μg/mL, it was not diluted to a volume equivalent to that of IV dexmedetomidine during IN administration. Fourth, the depth of anesthesia was assessed based on clinical evaluation other than bispectral index value. We cannot exclude that the sedation/anesthesia level was sufficient or even already high enough without the administration of dexmedetomidine. Therefore, further studies, particularly multicenter clinical studies, are required for optimization.

## Conclusion

Our results demonstrated that both IV and IN administration of dexmedetomidine after induction of anesthesia effectively improved emergence delirium and pain intensity in children undergoing adenotonsillectomy. Notably, IN administration of dexmedetomidine at 2 μg/kg after anesthesia induction offers advantages in terms of longer sedation and analgesia than the IV route. Moreover, IN administration of dexmedetomidine was associated with a lower incidence of hemodynamic instability than IV administration. With its more convenient and painless properties, the IN route of dexmedetomidine administration may foster strategies for the prevention of ED and postoperative pain in pediatric surgery. However, the optimal dosage and pharmacology of IN dexmedetomidine require further research.

## Data Availability

The datasets presented in this study can be found in online repositories. The names of the repository/repositories and accession number(s) can be found below: https://data.mendeley.com/datasets/xp7k6j78cp/1.

## Abbreviations

IV, intravenous; IN, intranasal; IV DEX, the intravenous dexmedetomidine group; IN DEX, the intranasal dexmedetomidine group; PAED, pediatric anesthesia emergence delirium; PACU, post-anesthesia care unit; ED, emergence delirium; CONSORT, Consolidated Standards of Reporting Trials; BMI, body mass index; PSAS, Parental Separation Anxiety Scale; mCHEOPS, modified Children’s Hospital of Eastern Ontario Pain Scale; MAP, mean arterial pressure; HR, heart rate; SD, standard deviation; ANOVA, analysis of variance; IQR, interquartile range; CI, confidence interval.
