# Supplementary material for: Comparative evaluation of intravenous versus intranasal dexmedetomidine on emergence delirium and hemodynamics in pediatric patients undergoing adenotonsillectomy: a randomized controlled trial
Source: Front Pharmacol. 2025 Jan 30;16:1543344. doi: 10.3389/fphar.2025.1543344 (PMC11821972; doi:10.3389/fphar.2025.1543344)
Supplement: Supplementary file 1 [file Table1.docx]

Supplementary Material

Supplementary Table 1. The Pediatric Anesthesia Emergence Delirium (PAED) scale

| Items |  |
| --- | --- |
| 1 | The child makes eye contact with the caregiver |
| 2 | The child’s actions are purposeful |
| 3 | The child is aware of his/her surroundings |
| 4 | The child is restless |
| 5 | The child is inconsolable |

Items 1, 2 and 3 are reversed scored as follows: 4 = not at all, 3 = just a little, 2 = quite a bit, 1 = very much, 0 = extremely. Items 4 and 5 are scored as follows: 0 = not at all, 1 = just a little, 2 = quite a bit, 3 = very much, 4 = extremely. The scores of each item were summed to obtain a total Pediatric Anesthesia Emergence Delirium (PAED) scale score. The degree of emergence delirium increased directly with the total score.

Supplementary Table 2. Ramsay Sedation Scale

| Score | Definition |
| --- | --- |
| 1 | Anxious and agitated or restless or both |
| 2 | Cooperative, oriented, and tranquil |
| 3 | Responds to commands only |
| 4 | Brisk response to a light glabellar tap or loud auditory stimulus |
| 5 | Sluggish response to a light glabellar tap or loud auditory stimulus |
| 6 | No response to a light glabellar tap or loud auditory stimulus |

Performed using a series of steps: observation of behavior (score 1 or 2), followed (if necessary) by assessment of response to voice (score 3), followed (if necessary) by assessment of response to loud auditory stimulus or light glabellar tap (score 4 to 6).

Supplementary Table 3. Modifed Children’s Hospital of Eastern Ontario Pain Scale (m-CHEOPS)

| Parameters | 0 | 1 | 2 |
| --- | --- | --- | --- |
| Crying | None | Crying moaning | Screaming |
| Facial expression | Smiling | Neutral | Grimacing |
| Verbal expression | Positive | None or another complaint | Complaining of pain |
| Torso | Neutral | Squirming, tense, upright | Restrained |
| Legs | Neutral | Kicking, restless, fetal position | Restrained |

Supplementary Table 4. The standards for modifed Aldrete scores

| Items | Standards | Scores |
| --- | --- | --- |
| Movement | Moving arms, legs and head spontaneously or by request; | 2 |
|  | Moving arms or legs spontaneously or by request, restrictedly raising head spontaneously or by request; | 1 |
|  | Not able to move limbs or raise head | 0 |
| Breathing | Deep breathing and effective coughing, normal respiratory rate and amplitude; | 2 |
|  | Breathing is difficult or restricted, and spontaneous breathing is shallow and slow, but it is possible to breathe through oropharyngeal airway; | 1 |
|  | Breathing is paused or weak, it requires a respirator therapy or assisted breathing | 0 |
| Blood pressure | Within ± 20% before anesthesia; | 2 |
|  | ± 20-49% before anesthesia; | 1 |
|  | Above ± 50% before anesthesia | 0 |
| Consciousness | Completely awakening, answer questions accurately; | 2 |
|  | Able to wake up, drowsiness; | 1 |
|  | No reaction | 0 |
| SpO_2_ | Air breathing SpO_2_ >92%; | 2 |
|  | Oxygen breathing SpO_2_ >92%; | 1 |
|  | Oxygen breathing SpO_2_ <92% | 0 |
